# BRAF Inhibitors Induce Feedback Activation of RAS Pathway in Thyroid Cancer Cells

**DOI:** 10.3390/ijms22115744

**Published:** 2021-05-27

**Authors:** Elisa Bonaldi, Chiara Gargiuli, Loris De Cecco, Arianna Micali, Maria Grazia Rizzetti, Angela Greco, Maria Grazia Borrello, Emanuela Minna

**Affiliations:** 1 Molecular Mechanisms Unit, Department of Research, Fondazione IRCCS Istituto Nazionale dei Tumori, 20133 Milan, Italy; eli.bon89@gmail.com (E.B.); mariagrazia.rizzetti@istitutotumori.mi.it (M.G.R.); Angela.Greco@istitutotumori.mi.it (A.G.); 2 Platform of Integrated Biology, Department of Applied Research and Technology Development, Fondazione IRCCS Istituto Nazionale dei Tumori, 20133 Milan, Italy; chiara.gargiuli@istitutotumori.mi.it (C.G.); loris.dececco@istitutotumori.mi.it (L.D.C.); arianna.micali@istitutotumori.mi.it (A.M.)

**Keywords:** thyroid cancer cell, *BRAF^V600E^*, BRAF inhibitors, cell signaling

## Abstract

*BRAF^V600E^* is the most frequent oncogenic mutation identified in papillary thyroid cancer (PTC). In PTC patients who do not respond to standard treatment, BRAF inhibitors are currently tested as alternative strategies. However, as observed for other targeted therapies, patients eventually develop drug resistance. The mechanisms of BRAF inhibitors response are still poorly understood in a thyroid cancer (TC) context. In this study, we investigated in *BRAF^V600E^* mutated TC cell lines the effects of Vemurafenib and Dabrafenib, two BRAF inhibitors currently used in a clinical setting. We assessed cell proliferation, and the expression and activity of the thyroid function related transporter NIS following the treatment with BRAF inhibitors. In addition, we investigated the global gene expression by microarray, the relevant modulated biological processes by gene set enrichment analysis (GSEA), and TC specific gene signatures related to MAPK pathway activation, thyroid differentiation, and transcriptional profile associated with *BRAF^V600E^* or *RAS* mutation. We found that both inhibitors induce antiproliferative and redifferentiative effects on TC cells, as well as a rewiring of the MAPK pathway related to RAS signaling. Our results suggest a possible mechanism of drug response to the BRAF inhibitors Vemurafenib or Dabrafenib, supporting very recent findings in TC patients treated with targeted therapies.

## 1. Introduction

Thyroid cancer (TC) is the most common neoplasia of the endocrine system and comprises various histological types including, among others, the well differentiated papillary and follicular thyroid carcinoma (PTC and FTC, respectively) and the undifferentiated anaplastic thyroid carcinoma (ATC), which collectively account for more than 90% of all thyroid tumors [1].

Molecular studies aimed at the dissection of the genetic players involved in thyroid carcinogenesis have led to the identification of several tumor-driving events, many of which consist in mutually exclusive alterations of genes encoding effectors of the mitogen activated protein kinase (MAPK) pathway. Among these, *BRAF^V600E^* is the most frequently detected mutation, followed by *RAS* mutations, and chromosomal rearrangements of tyrosine kinase receptors (such as *RET*, *NTRK*, and *ALK*) [2]. A significant association among different driver mutations and histological types of thyroid carcinomas has been identified; *BRAF^V600E^*, for instance, is detected at high frequency in PTC (60–74%, [3,4]) and ATC (41–45% [4,5]), in this latter often in combination with other alterations (such as *TP53* and *TERT* promoter mutations), while it is rare in FTC, where *RAS* mutations are more frequent (66% [4,6]).

Along with the molecular studies, more recently, transcriptomic analyses have led to the identification of distinct gene expression profiles associated with specific TC histotypes, as well as with PTC histological variants, driving lesions and clinical features. In this context, a milestone was represented by the work from the Cancer Genome Atlas (TCGA) on a large cohort of PTCs [3], where the authors reported for the first time three gene signatures able to describe distinct but interconnected features of thyroid tissue, such as: (i) the activation status of the MAPK pathway (MAPK output); (ii) the degree of thyroid differentiation (TD); and (iii) the presence of a transcriptional profile related to *BRAF^V600E^* or *RAS* mutation (BRAF–RAS signaling). By this approach, the authors highlighted how *BRAF^V600E^* mutated PTCs display concurrent BRAF signaling, increased activation of MAPK pathway, and reduced expression of thyroid function related genes, which include genes involved in iodide transport, organification, and incorporation into thyroglobulin to produce thyroid hormones.

These TCGA derived gene signatures have been subsequently applied and validated by multiple independent studies on TCs [5,6,7,8,9,10], and more recently, also on TC derived cell lines [11] and on patient derived biopsies collected before and after BRAF inhibitor treatment [12].

The vast majority of thyroid tumors are PTCs (80–85%), which represent the prevalent histotype. Most PTCs are clinically indolent diseases, effectively treated with surgical removal alone or, for high risk cases, followed by radioactive iodine (RAI) treatment [13]. The specific use of RAI is based on the intrinsic ability of thyroid follicular cells to uptake iodine (necessary for the physiological synthesis of thyroid hormones) by the transporter sodium–iodine symporter (NIS) [14]. The ability of thyroid cells to uptake and accumulate iodine, and therefore RAI, is a critical factor for the clinical effectiveness of RAI therapy [15]. Indeed, in a fraction of patients defined as RAI-resistant, and characterized by low or absent RAI uptake and/or disease persistence or progression following RAI treatment, the prognosis is poor. In these high risk patients who do not respond to RAI [16], the alternative treatment with systemic therapies based on BRAF or MEK inhibitors, tested as a single agent [17,18,19] or in combination (reviewed in [20]), has been introduced. Along with the anti-tumoral effect, more recently these targeted therapies have also been tested for the possibility of restoring radioiodine sensitivity in RAI-resistant thyroid cancers, and thus, of being exploited as a redifferentiation strategy.

The investigations of redifferentiation strategies have resulted in particular interest especially for BRAF mutated PTCs that display more frequently RAI refractoriness and poor response [1,9,21]. In these tumors, the RAI refractoriness appears to be due to the high MAPK-pathway output driven by the BRAF^V600E^ oncoprotein [2], which suppresses the expression of the thyroid function related genes involved in iodide-handling machinery.

Starting from the observation that different MAPK inhibitors are able to restore NIS expression and/or iodine uptake in various in vitro [20] and in vivo models [22] of thyroid cancer, several studies focused on redifferentiation strategies have been undertaken in a clinical setting (reviewed in [23]). However, limited and variable results were obtained, with RAI uptake restoration only in a fraction of patients and clinical response ranging from partial to null, possibly due to the activation of alternative signaling pathways leading to drug resistance.

In this study, to better understand the molecular processes involved in BRAF inhibitors drug response, we investigated in vitro the two BRAF inhibitors Vemurafenib and Dabrafenib on *BRAF^V600E^* mutated thyroid cancer cell lines. We tested their effects on cell growth and as redifferentiation strategies, as well as on transcriptomic profiles.

## 2. Results

### 2.1. BRAF^V600E^ Mutation and Reduced NIS Expression Are Found in Thyroid Cancer Cell Lines

**Figure 1:**
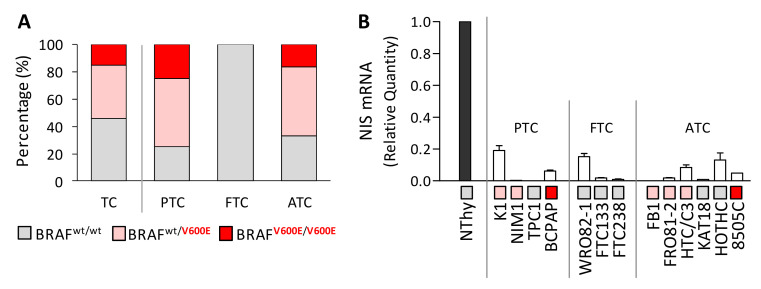
*BRAF^V600E^* mutation and *NIS* expression in thyroid cancer cell lines. (**A**) *BRAF^V600E^* distribution in TC cell lines and in the same cell panel stratified according to tumor histotype. Complete *BRAF* mutation data available in Suppl. Table S1. (**B**) *NIS* expression by quantitative RT-PCR; data are reported as relative quantity normalized to *HPRT* gene used as endogenous control for RNA input normalization and are shown relative to the control cell line Nthy; mean ± SEM of three technical replicates. For each cell line, the *BRAF* mutational status is indicated with a color code legend as in (**A**). Abbreviations: TC, Thyroid Cancer; PTC, Papillary Thyroid Cancer; FTC, Follicular Thyroid Cancer; ATC, Anaplastic Thyroid Cancer; wt, wild-type.

We profiled a panel of thyroid cancer cell lines for the presence of *BRAF^V600E^* mutation, including 13 TC cell lines derived from different thyroid tumor histotypes, and Nthy control cells derived from non-neoplastic thyroid (NT) (Table S1). *BRAF^V600E^* mutation was investigated by *BRAF* exon 15 PCR followed by automated Sanger sequencing and confirmed by mutant allele specific amplification (MASA)-PCR (Figure S1). In agreement with previous reports (Table S1), the presence of *BRAF^V600E^* was confirmed in a high fraction of the tested TC cells (67%), both in homo and in heterozygosis (Figure 1A), while as expected, it was absent (*BRAF* wt) in the control cells Nthy. Consistently with the *BRAF^V600E^* distribution in human TC tissues, *BRAF^V600E^* was found in TC cells derived from PTC and ATC tissues, while it was absent in the FTC derived cell lines (Figure 1A and Table S1).

In the same cell panel, we then assessed the expression of *NIS* and confirmed its reduced or undetectable level in TC cells compared to the NT control Nthy (Figure 1B), according with the NIS low expression reported in human TC tissues and in TC cell lines [11,24].

### 2.2. BRAF Inhibitors Affect Cell Proliferation, NIS Expression, and Activity in BRAF^V600E^ Mutated Thyroid Cancer Cell Lines

**Figure 2:**
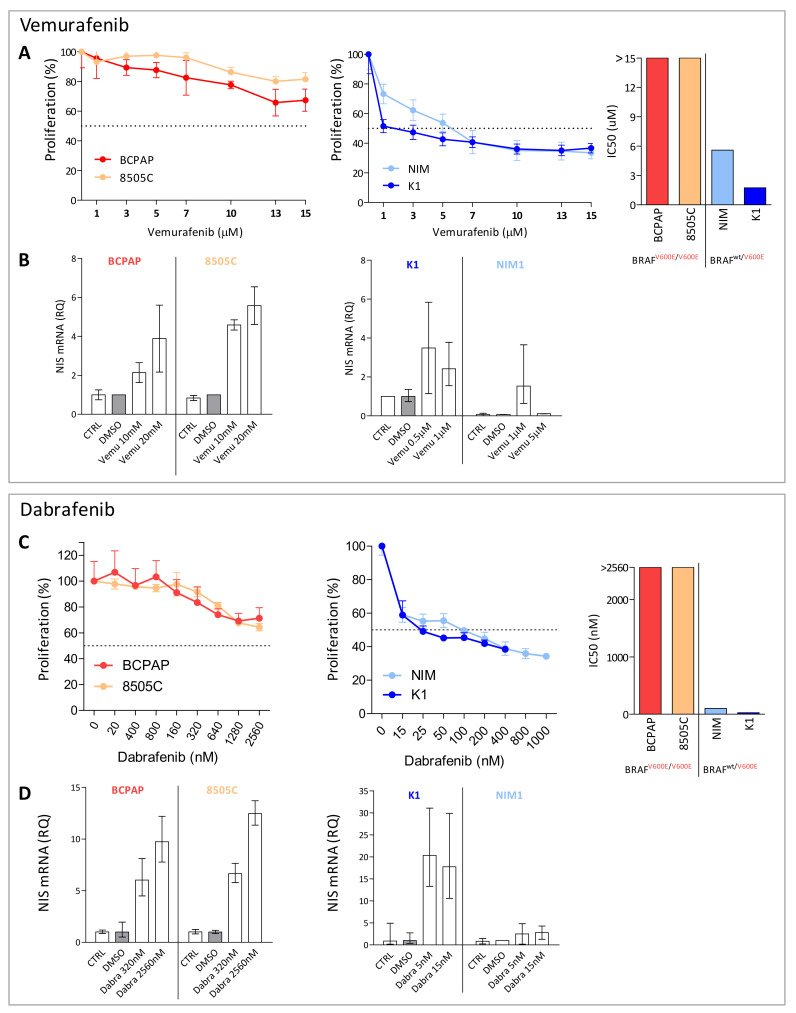
BRAF inhibitors treatment in *BRAF^V600E^* mutated thyroid cancer cell lines. (**A**,**C**) Dose-response curves in thyroid cancer cell lines (BCPAP, 8505C, K1, NIM1) treated for 48 h with increasing doses of Vemurafenib or Dabrafenib. Cell proliferation was tested by crystal violet assay; data are reported as percentage normalized to control cells treated with vehicle DMSO, mean ± SD of six technical replicates. On the right, the corresponding IC50 values in the same cell lines stratified for *BRAF^V600E^* mutation status. (**B**,**D**) *NIS* expression by quantitative RT-PCR 48 h after Vemurafenib or Dabrafenib treatment; data are reported as relative quantity normalized to *HPRT* gene used as endogenous control and are shown relative to the control cells treated with DMSO. Mean ± SEM of three technical replicates. Abbreviations: CTRL, untreated control cells; DMSO, DMSO treated control cells; Vemu, Vemurafenib; Dabra, Dabrafenib.

We then tested the response to the BRAF selective inhibitors Vemurafenib or Dabrafenib in four *BRAF^V600E^* mutated TC cell lines (BCPAP, 8505C, K1, and NIM1). In all the tested cell lines, we observed proliferation reduction in a dose dependent manner following treatment (Figure 2A,C) and drug response was correlated with *BRAF* mutational status, displaying higher sensitivity in TC cells with heterozygous *BRAF^V600E^* than in those with homozygous *BRAF^V600E^* (Vemurafenib IC50 1.7 µM and 5.6 µM in K1 and NIM1, and >15 µM in both BCPAP and 8505c; Dabrafenib IC50 25 nM and 100 nM in K1 and NIM1, and >2.56 µM in both BCPAP and 8505C).

Based on drug response results, we selected two BRAF inhibitors’ doses and, in addition to proliferation, we also investigated *NIS* levels (Figure 2B,D); increased *NIS* expression was detected following both treatments.

**Figure 3:**
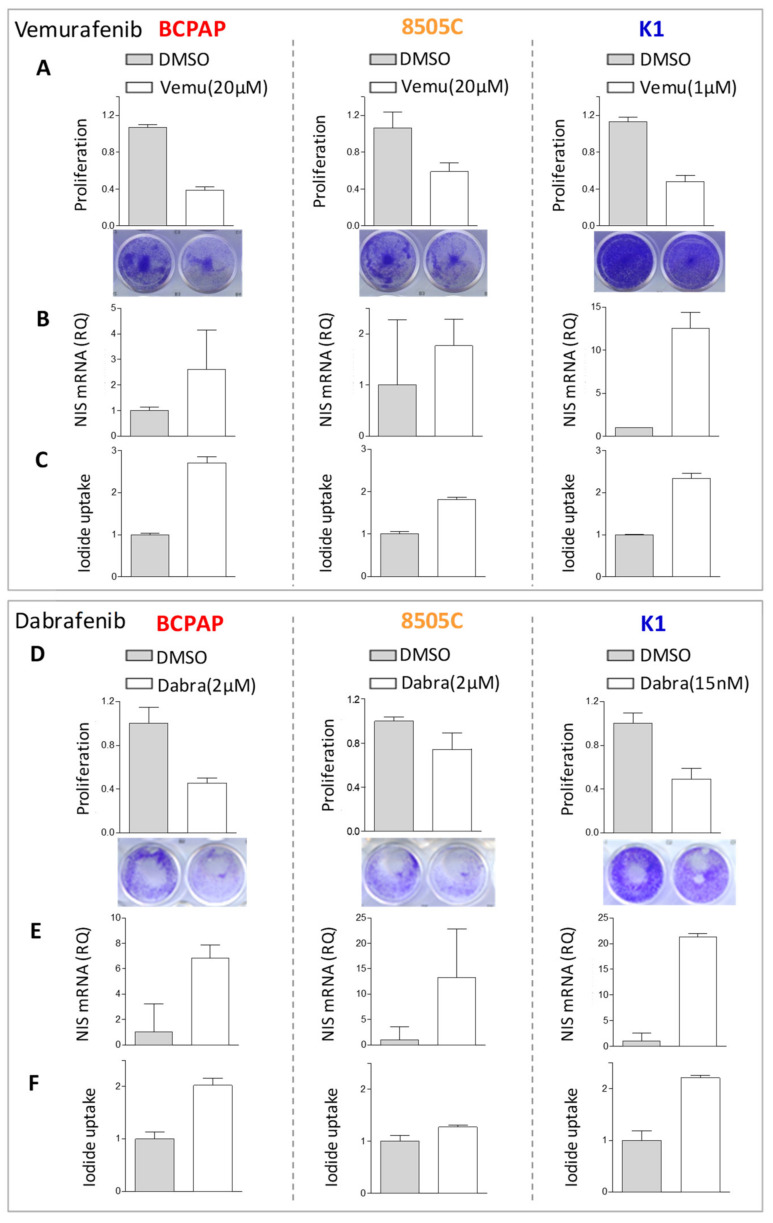
Iodide uptake capacity in thyroid cancer cells following BRAF inhibitors treatment. (**A**,**D**) Cell proliferation by crystal violet assay in DMSO and BRAF inhibitors treated cells; data are recorded as absorbance at 570 nm and are normalized to control cells treated with vehicle DMSO; mean ± SD of technical replicates. Below representative images of crystal violet stained cell culture plates. (**B,E**) *NIS* expression by quantitative RT-PCR in DMSO and BRAF inhibitors treated cells; data are reported as relative quantity normalized to HPRT gene used as endogenous control and are shown relative to DMSO treated control cells; mean ± SEM of three technical replicates. (**C**,**F**) Iodide uptake assay by Sandell–Kolthoff reaction, absorbance detected at 420 nm. Uptake value was normalized to the corresponding cell proliferation index reported above; data are reported relative to the DMSO treated control cells as mean ± SD of technical replicates. All assessments were performed at 48 h after BRAF inhibitors treatment. Abbreviations: DMSO, DMSO treated control cells; Vemu, Vemurafenib; Dabra, Dabrafenib.

In the cell lines showing consistent *NIS* increase, we also observed a concurrent increase of iodide uptake capacity in treated cells (Figure 3C,F), even though variable across the tested cell lines and of moderate entity. We then focused on BCPAP cell line, displaying a higher and consistent increase of iodide uptake across the two BRAF inhibitor treatments, and investigated its transcriptional profiles.

### 2.3. BRAF Inhibitors Induce Gene Expression Modulation and RAS Pathway Activation

**Figure 4:**
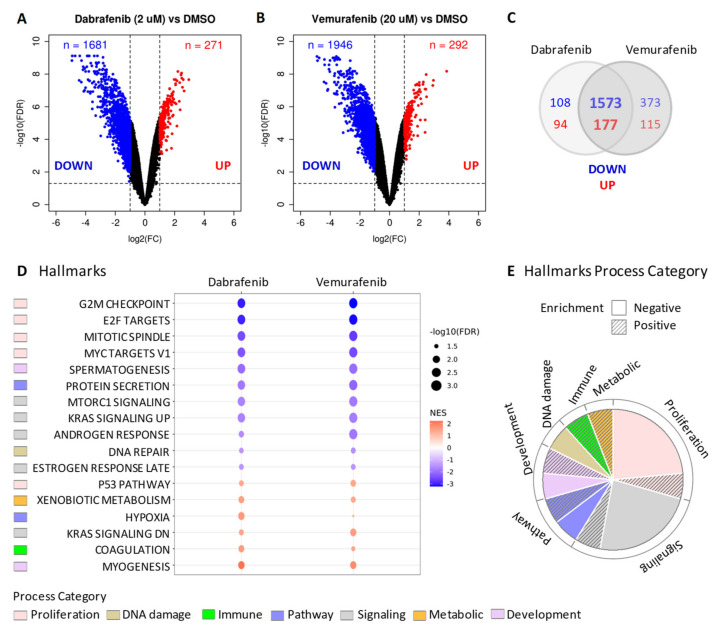
Gene expression profiles in BCPAP cells treated with BRAF inhibitors. (**A**,**B**) Volcano plot of differentially expressed genes between either Dabrafenib or Vemurafenib treated BCPAP cells compared to control cells treated with DMSO. The *x*-axis shows the log2 fold change (FC) and the *y*-axis shows the −log10 false discovery rate (FDR). The vertical and horizontal dashed lines represent the FC and FDR thresholds (absolute FC > 2 and FDR < 0.05, respectively) used to select differentially expressed genes. Up- and down-regulated genes in each comparison are highlighted in red and blue, respectively. (**C**) Venn diagram showing the intersection of statistically significant deregulated genes selected with cutoffs on absolute FC > 2 and FDR < 0.05. (**D**) Dot plots showing commonly significantly enriched gene sets (FDR < 0.05) from GSEA Hallmarks collection. The red-to-blue colored-scale bar represents the normalized enrichment score (NES). The point size indicates the magnitude of the statistical significance expressed as –log10 of the FDR. (**E**). Distribution of Hallmarks process categories relative to gene sets showed in (**D**).

Gene expression profiles were established by microarray in BCPAP cells treated with Vemurafenib (20 µM) or Dabrafenib (2 µM) compared to control cells treated with vehicle DMSO. Significant gene deregulation was found in BRAF inhibitors treated cells compared to control (Figure 4A,B). Gene downregulation was prevalently observed, identifying a broad set of genes with significantly reduced expression following the treatments (Figure 4A,B, FDR < 0.05 and Fold Change < −2; 1681 and 1946 significant downregulated genes in Dabrafenib and Vemurafenib, respectively). Consistent results were obtained across the two treatments, with most of differentially expressed genes commonly and concordantly modulated (Figure 4C).

To dissect the biological processes and pathways associated with the identified gene deregulation, we then performed gene set enrichment analysis (GSEA) using the Hallmark gene set collection. Several pathways were enriched in treated cells compared to DMSO (FDR < 0.05, Figure S2), and the most significant were associated with gene downregulation (negatively enriched pathways). The top enriched pathways were concordant across the two treatments (Figure 4D) and were mainly related to the proliferation and signaling category (Figure 4E), in accordance with the reduced proliferation phenotype observed upon BRAF pharmacological inhibition (Figure 2A,C and Figure 3A,D). The most impaired pathways (negative NES, Figure 4D) were related to cell cycle progression, including G2/M checkpoint, mitotic spindle assembly, cell cycle related targets of E2F transcription factor, and genes regulated by MYC; a reduction in DNA damage response was also observed. Among positively enriched pathways (mediated by genes upregulation), we identified processes activated in stress conditions such as, among others, hypoxia, genes involved in metabolism of drugs and xenobiotics, consistent with a drug treatment, and activation of the p53 pathway.

Of note, we also found the significant enrichment of two gene sets termed KRAS signaling (i.e., KRAS_SIGNALING_UP and KRAS_SIGNALING_DN), that include genes upregulated and downregulated, respectively, upon activation of oncogenic KRAS [25]. Collectively, they describe the gene modulation induced by the oncogenic activation of this effector belonging to the MAPK pathway. Transcriptional activation (upregulation of KRAS_SIGNALING_UP genes and downregulation of KRAS_SIGNALING_DN genes) was detected in control cells, consistently with the presence of an active oncogenic BRAF^V600E^ (Figure S3A). Instead, in treated cells, where BRAF^V600E^ oncogenic signaling was inhibited by Vemurafenib or Dabrafenib, we observed an opposite expression pattern (Figure S3A) with KRAS_SIGNALING_UP negatively enriched (negative NES) and KRAS_SIGNALING_DN positively enriched (positive NES) (Figure S3B and Figure 4D), suggesting transcriptional modulation of this set of genes related to the MAPK pathway following BRAF inhibitors’ treatment.

An analogous transcriptional modulation was detected by testing the three gene signatures previously reported by TCGA in the specific context of thyroid cancer [3] and related to MAPK output, thyroid differentiation (TD), and transcriptional profile associated with *BRAF^V600E^* or *RAS* mutation (BRAF-RAS signaling). For each of these signatures, we also calculated the corresponding score summarizing the multiple genes information in a single variable.

**Figure 5:**
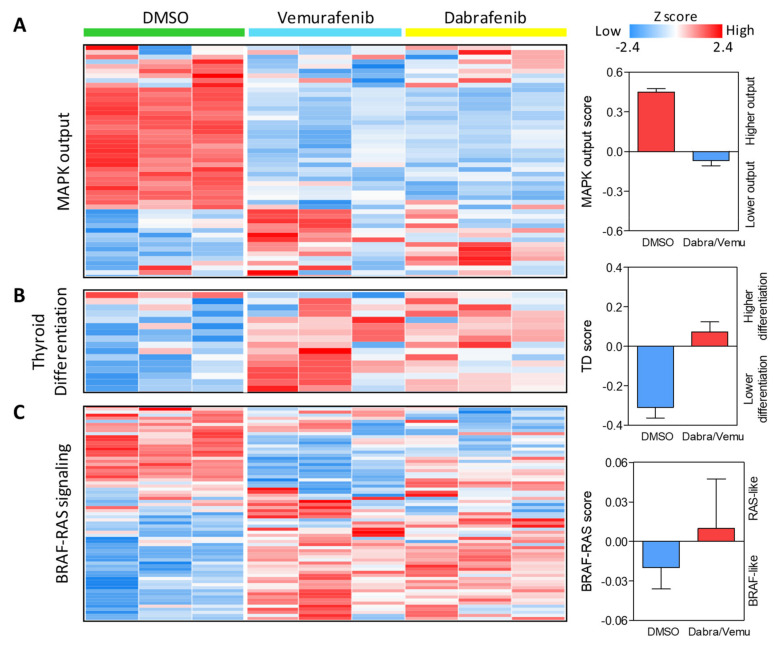
Expression of thyroid cancer related gene signatures in BRAF inhibitor treated BCPAP cells. (**A**) Gene signature related to the activation status of MAPK pathway (MAPK output). (**B**) Gene signature related to thyroid differentiation. (**C**) Gene signature related to BRAF-RAS signaling. The 3 gene signatures are derived from TCGA, lists available in Supplementary Table S2. Heatmaps show the expression of the genes included in each signature across control (DMSO) and BRAF inhibitor (Vemurafenib or Dabrafenib) treated cells in 3 technical replicates per condition. On the right, the corresponding expression score (see Materials and methods for score computation).

In agreement with GSEA Hallmarks results, in treated cells, we found reduced expression of MAPK pathway genes and a lower MAPK output score compared to control cells (Figure 5A). In addition, consistently with the NIS enhanced expression and activity observed upon BRAF pharmacological inhibition (Figure 3), in treated cells, we also detected upregulation of other thyroid function related genes and a higher degree of thyroid differentiation (Figure 5B).

When we assessed the BRAF-RAS signaling, in control cells we observed a BRAF-like profile, in accordance with the presence of an active BRAF^V600E^, while in treated cells, we observed an opposite expression pattern and a RAS-like profile (Figure 5C), indicative of an alternative activation of the MAPK pathway.

**Figure 6:**
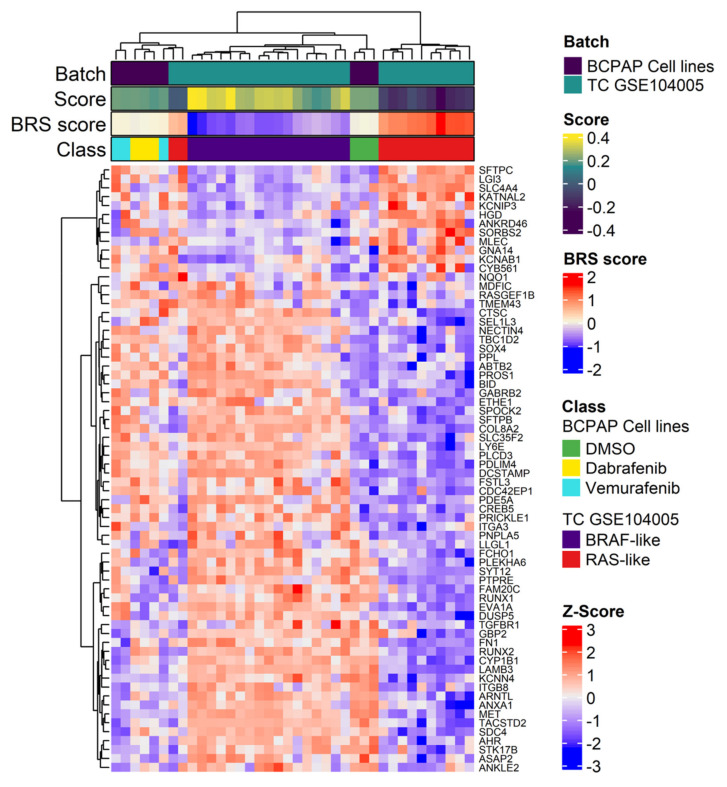
Unsupervised hierarchical clustering using the BRAF-RAS signaling gene signature in BCPAP cells and in thyroid cancer tissues. Heatmap showing the expression pattern of the genes, reported as Z-scores, included in the BRAF-RAS like signature evaluated in the merged datasets, including the BCPAP cells and the human thyroid cancer (TC) tissues derived from the gene dataset GSE104005. Specific sample features are showed separately in the colored bar above the heatmap: Batch, either BCPAP cell lines or GSE104005 samples; Score, BRAF−/RAS-like subtype gene scores calculated with singscore; BRS, BRAF-RAS score; Class, BCPAP cell lines stratified for treatment; TC tissues from GSE104005 dataset stratified for BRAF/RAS subtype; Z-Score, colored bar z-scores values.

This was further confirmed by comparing the gene expression profiles between BCPAP cells and human thyroid cancer tissues (series GSE104005) previously established by our laboratory and classified for the BRAF- or RAS-like signaling subtype [8]. In unsupervised hierarchical clustering (Figure 6), BCPAP control samples (DMSO) grouped together with BRAF-like TCs, while Dabrafenib and Vemurafenib treated cells grouped in a separate cluster with some RAS-like TCs. Similar results were obtained by principal component analysis (PCA), where Dabrafenib and Vemurafenib treated cells grouped closer to RAS-like TC tissues than BCPAP control cells (Figure S4).

Interestingly, by assessing the expression of genes included in BRAF-RAS signature, in both control and treated BCPAP cells, we observed more deregulated expression patterns and reduced levels of BRAF-RAS score (BRS) values than those observed in GSE104005 TC tissues (Figure 6), most of which consist of well differentiated thyroid cancers, as previously described [8]. This is consistent with the notion that in thyroid cancer cell lines [11], as well as in advanced thyroid cancers [5], the BRAF-/RAS-like classification is preserved but the two subtypes exhibit less profound differences and reduced BRS values. This observation is in agreement with the already reported findings that thyroid cancer cell lines, due to in vitro culture selection, display some dedifferentiation features common to advanced thyroid tumors [11,24].

**Figure 7:**
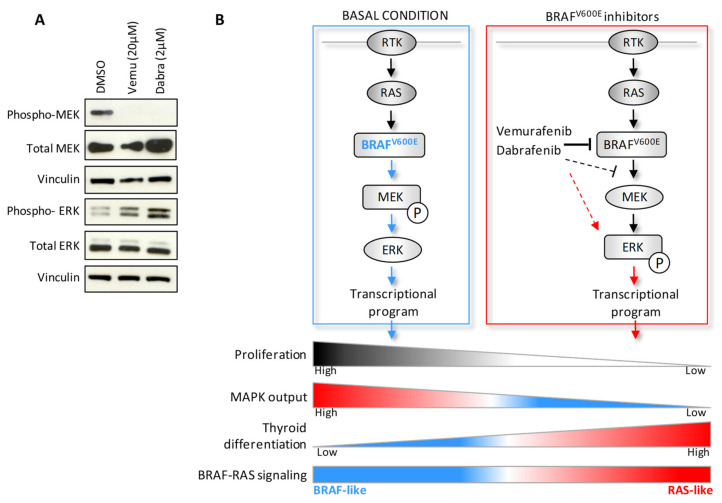
Alternative MAPK pathway activation upon treatment with BRAF inhibitors in BCPAP cells. (**A**) Western blot analysis in BCPAP cells treated with Vemurafenib or Dabrafenib for 48 h compared to control cells treated with vehicle DMSO. Vinculin included as protein loading control. (**B**) Schematic representation of the expression pattern identified by transcriptomic and biochemical analyses in BCPAP cells treated with BRAF inhibitors.

The alternative activation of the MAPK pathway identified by transcriptomic data was also verified at protein level. In BCPAP cells treated with Vemurafenib or Dabrafenib, we detected not only the impairment of BRAF^V600E^ signaling on its downstream effector MEK, by the abrogation of its phosphorylation, but also a feedback activation by phosphorylation of ERK (Figure 7A). Similar effects were confirmed in other *BRAF^V600E^* mutated TC cell lines treated with the two BRAF inhibitors (Figure S5).

## 3. Discussion

In this study, we investigated the molecular processes associated with drug response to BRAF inhibitors in *BRAF^V600E^* mutated thyroid cancer cell lines. Along with the antiproliferative and redifferentiative effects, we found that the pharmacological inhibition of BRAF^V600E^ induces an alternative feedback activation of the MAPK pathway related to RAS signaling, detected at transcriptomic and protein level (Figure 7B).

The identification of genetic effectors driving tumor pathogenesis has led to the development of selective compounds targeting these specific genetic alterations, opening the era of molecular targeted therapies. BRAF inhibitors are included in this category of new therapeutic compounds. Starting from their primary application in melanoma cancer patients with *BRAF^V600E^* mutation, in which high efficiency in tumor growth inhibition had been initially observed [26,27], the possibility of exploiting BRAF inhibitors has been extended also to thyroid cancer patients. In these latter, BRAF targeting compounds have been demonstrated to reduce tumor growth, showing efficacy in a fraction of *BRAF*-mutated PTC [17] and ATC [28]. This has led to the FDA approval of Vemurafenib and Dabrafenib for the treatment of *BRAF^V600E^* advanced RAI-refractory thyroid cancer and metastatic PTC, respectively [23], and of the combinatorial regimen Dabrafenib plus Trametinib in ATC patients.

In the present study, we confirmed the antiproliferative effect of both BRAF inhibitors Vemurafenib and Dabrafenib in agreement with previous reports.

In addition to the antitumoral effect, in the specific context of TC, the identification of reinduced expression of genes related to thyroid function and of RAI uptake capacity has raised the possibility of the use of BRAF inhibitors as a redifferentiation strategy. However, even though several attempts have been done for this purpose, limited efficiency has been observed. Variable or in some instances disappointing results have indeed been obtained, as for Selumetinib. Whereas the initial study of this compound represented the proof-of-concept for the redifferentiation therapy [19] and had raised a renewed interest about this topic, the subsequent phase 3 trial in combination with RAI did not improve outcome for patients with differentiated thyroid cancer [29]. Additionally, for BRAF inhibitors, the investigations of RAI uptake restoration have produced variable results as demonstrated in TC patients, where only a fraction of patients displayed restored or new RAI uptake after treatment [20,23]. Here, in agreement with clinical data, we observed also in our cell models reinduced expression of the iodide transport gene *NIS* and of other genes related to thyroid function, as well as an increase in the iodide uptake capacity, but this latter effect is variable across the tested cell lines and of modest intensity. Thus, the application of BRAF inhibitors as redifferentiation therapies remains to be confirmed and/or optimized by more in depth studies.

Despite these limitations as redifferentiation strategies, BRAF targeting therapies have showed antitumoral efficacy in a subset of TCs [23]. Their use, however, still represents a clinical challenge, as patients display nondurable response and frequently develop drug resistance. Several mechanisms of acquired resistance following BRAF/MEK inhibitor treatment have been identified in other types of *BRAF^V600E^* mutated tumors, such as melanoma and colorectal cancer involving, for instance, either the reactivation of the MAPK pathway, or activation of parallel signaling cascades such as the PI3K/AKT pathway, or acquired mutations of the *RAS* gene family [30,31,32].

By contrast, in thyroid cancer, this still represents a poorly understood and explored field. This is in part due to the limited availability of advanced thyroid cancer tissue specimens for deep molecular testing, and in particular, those from patients with drug resistant, progressive and/or recurrent disease. The major factors at the base of these specimens’ inaccessibility are the relatively low frequency of aggressive or non-responding TC cases, and the progressively reduced number of surgical interventions. These latter, according to the most recent guidelines [33], are indeed recommended only in the fraction of patients with rapidly progressive and symptomatic disease or with high risk of local complications, or result not applicable in widespread or unresectable tumors (frequently observed in ATC cases).

To overcome this problem, the investigations of the BRAF inhibitor drug response have been performed mainly by preclinical studies in TC cell line models. By this approach, some possible mechanisms responsible for drug resistance have been proposed, involving, for instance, either the over expression of the HER3 receptor [34,35] or activation of other players [36,37], often associated with rebound activation of phospho-ERK [34,35,38,39], as observed in the present study and in melanoma cells [40].

However, most of these in vitro studies of TC models investigated a limited number of genes and/or effectors and only a few of the proposed drug response mechanisms have been confirmed in resistant TC patients.

In this study, we assess by a high throughput analysis the global gene expression profiles of thyroid cancer cells treated in parallel with two BRAF inhibitors. By this approach, we identified in the BRAF inhibitor treated cell a rewiring of the MAPK pathway associated with RAS signaling.

Consistently with our data, two recent studies reported the acquisition of secondary *RAS* gene mutations in thyroid cancer patients treated with BRAF inhibitors. The first is a case report in which has been shown for the first time the acquisition of a *RAS* mutation (*KRAS^G12V^*) in a patient with *BRAF* mutated PTC during BRAF/MEK inhibitors therapy [41]. The second study describes four *BRAF^V600E^*-mutated thyroid cancer patients with disease progression during BRAF or BRAF/MEK inhibitor treatment and acquisition of secondary mutations in *RAS* genes (*KRAS^G12V^* in two patients, and *NRAS^Q61K^* and *NRAS^G13D^* in the others) [42]. Interestingly, in this second study, the authors overcome the inaccessibility of tumor material for molecular testing for some patients by assessing the mutational load in circulating DNA on patient liquid biopsies, highlighting how additional more advanced techniques are required for this type of analyses in thyroid cancer context.

In agreement with these reports in TC patients, the development of drug resistance mediated by the emergence of an additional *RAS* mutation (*KRAS^G12V^*) has been previously demonstrated in a *BRAF* mutated PTC cell line treated with Vemurafenib [39]. Of note, in our study, we identified a novel RAS-mediated mechanism of drug resistance associated with the transcriptional modulation of a signaling pathway related to *RAS* gene activation, defined RAS-like profile, that is commonly shared by different types of *RAS* mutations, as confirmed by TCGA [3] and other studies [5,8] showing that various driving mutations affecting *RAS* genes (*NRAS^Q61R^*, *HRAS^Q61R^*, *HRAS^Q61K^*, *KRAS^Q61R^*, *KRAS^Q61K^*, *KRAS^G12V^*) all consistently display a RAS-like profile.

A similar transcriptional activation was confirmed in serial biopsies from three TC patients treated with Vemurafenib [12]. In these patients has been reported not only the decrease of tumor lesion size and the reinduction of RAI uptake upon Vemurafenib treatment, but also by transcriptomic analyses a lower MAPK output, a RAS-like signaling, and higher thyroid differentiation compared to each patient’s paired basal condition before Vemurafenib administration.

Collectively, these findings from TC patients support the results here reported obtained in experimental models and their applicability in future studies aimed at a better dissection of the molecular processes associated with targeted therapy response. Indeed, the development of resistance to BRAF inhibitors tested as single agents has already led to the exploration of alternative therapeutic strategies, including combination of BRAF and MEK inhibitors and immune-modulating agents. However, only few data are currently available on drug efficacy and/or response, and further preclinical and clinical studies are required.

The results here described not only indicate an additional possible mechanism of BRAF inhibitor drug resistance, but also support the validity and utility of preclinical models, especially in the TC context where difficulties due to the inaccessibility of specimens from non-responding patients are progressively becoming more frequent.

## 4. Materials and Methods

### 4.1. Thyroid Cell Cultures

Thirteen human TC cell lines derived from different histotypes were investigated (Table S1); the Nthy-ori 3–1 cell line (Nthy; SV-40 immortalized normal human thyroid follicular cells) was included as non-neoplastic thyroid control. Cell lines were purchased from the commercial repositories European Collection of Cell Cultures (ECACC) or Riken Cell Bank; FB1 cells were obtained from its original establisher [43]. All cell lines were cultured in the recommended media supplemented with 10% (*v*/*v*) heat-inactivated fetal bovine serum (FBS, EuroClone, Pero, Italy) as monolayers at 37 °C in a 5% CO_2_ humidified atmosphere. Nthy were cultured in RPMI 1640 medium (Gibco, Thermo Fisher Scientific, Waltham, MA, USA); TPC1, NIM, BCPAP, WRO82-1, 8505C, KAT18, HTC/C3, FB1, and FRO81-2 were cultured in DMEM medium (Gibco); K1 were cultured in DMEM: Ham’s F12: MCDB (ratio 2:1:1); HOTHC were cultured in Ham’s F12; and FTC133 and FTC238 were cultured DMEM: Ham’s F12 (ratio 1:1).

Cell lines were authenticated by short tandem repeat (STR) profiles according to ATCC guidelines using GenePrint 10 System (Promega) including ten loci (TH01, TPOX, vWA, Amelogenin, CSF1PO, D16S539, D7S820, D13S317, D5S818, and D21S11); the obtained STR profiles matched with reference profiles (Table S1) confirming lineage identity. Cell lines were routinely tested for absence of mycoplasma contamination by PCR Mycoplasma Detection Set (TAKARA Bio Inc, Shiga, Japan).

All cell lines were subjected to total RNA extraction by NucleoSpin RNA isolation kit (Macherey-Nagel, Duren, Germany) and cDNA synthesis by SuperScript III First-Strand Synthesis System for RT-PCR (Invitrogen, Thermo Fisher Scientific).

### 4.2. BRAF^V600E^ Mutation Assessment

*BRAF* mutational analysis was performed on cDNA by standard PCR with *BRAF* exon 15 specific primers as previously described [44] followed by automated Sanger sequencing (Eurofins Genomics Services); sequences were evaluated by Chromas Lite software. Additional assessment was performed as previously described [45] by the highly sensitive mutant-allele-specific-amplification (MASA) PCR method for the specific detection of *BRAF^V600E^*, using a forward primer designed with two mismatches at the 3′ to amplify mutant *BRAF^V600E^* and a reverse primer derived from wild-type *BRAF* to amplify both wild-type and mutant sequences. PCR products were resolved by agarose gel electrophoresis.

### 4.3. Quantitative Real-Time Reverse Transcription PCR (qRT-PCR)

The expression of *NIS* (*SLC5A5* gene) was determined by a two-step quantitative real-time PCR on cDNA synthesized from total RNA. TaqMan Universal PCR Master Mix and Gene Expression Assay Hs00950356_m1 (*SLC5A5* gene) were used (Applied Biosystems, Thermo Fisher Scientific); *HPRT* gene (assay hs02800695_m1) was used as endogenous control for data normalization. All qRT-PCRs were performed in triplicate on the ABI PRISM 7900HT Real-Time PCR System. Data were analyzed with SDS 2.4 and RQ Manager 1.2.1 software (Applied Biosystems) using the 2^-∆∆Ct^ method.

### 4.4. Pharmacological Treatments and Cell Proliferation Assay

Vemurafenib (PLX4032) and Dabrafenib (GSK2118436) were purchased from Selleck Chemicals (Houston, TX, USA) and were used as BRAF^V600E^ selective inhibitors. Testing doses were initially selected from previous literature reports. Drugs were dissolved into DMSO and stored at −20 °C; working solutions were freshly prepared with cell culture medium at the moment of treatment. Cells were seeded in well culture plates 24 h before treatment; cell lines were treated with drugs increasing doses for 48 h. The effect of BRAF inhibitors on cell proliferation was assessed by crystal violet assay as previously described [46] and absorbance measured at 570 nm by a microplate reader (TecanUltra, Tecan Trading AG, Männedorf, Switzerland).

### 4.5. Iodide Uptake Assay

The NIS transporter activity was evaluated by a spectrophotometric iodine uptake assay optimized from the protocol by Waltz et al. [47] based on the catalytic effect of iodide on Sandell–Kolthoff reaction (reduction of yellow cerium(IV) to colorless cerium(III) in the presence of arsenious acid). BCPAP, 8505C, and K1 cells were treated with DMSO or Vemurafenib (20 or 1 µM) or Dabrafenib (2 µM or 15 nM) in two parallel culture plates and 48 h after treatment, the iodine uptake capacity was assessed. Briefly, the culture medium was removed and cells were washed with Hank’s balanced salt solution (HBSS, Euroclone) supplemented with 10 mM Hepes; cells were then incubated for 1 h at 37 °C (5% CO_2_ atmosphere) with uptake buffer consisting of HBSS/Hepes supplemented with 10 µM sodium iodide (Sigma-Aldrich, St. Louis, MO, USA) solution. Uptake buffer was removed and cells were washed with ice-cold HBSS/Hepes; one plate was subjected to iodide determination by Sandell–Kolthoff reaction and the other to cell proliferation index determination by crystal violet assay. For iodide determination, 10.5 mM ammonium cerium (IV) sulphate solution (Sigma-Aldrich) and 24 mM sodium arsenite (III) solution (Sigma-Aldrich) were added in each well and incubated at room temperature in the dark for 30 min; absorbance at 420 nm was recorded by a microplate reader (TecanUltra). The iodide concentration was determined by interpolation with a calibration curve prepared with iodide standards. Iodide concentration values were normalized on the corresponding cell proliferation index obtained in the parallel plate.

### 4.6. Gene Profiling

Gene expression profiles were established by microarray analysis in BCPAP cells treated with BRAF inhibitors (Vemurafenib 20 µM or Dabrafenib 2 µM) or with vehicle DMSO; three technical replicates per condition were assessed. Total RNA was extracted 48 h after treatment by miRNeasy mini kit (Qiagen) and quantified by Qubit 4 Fluorometer (Thermo Fisher Scientific); RNA quality was evaluated by the RNA Integrity Number (RIN) assessed by TapeStation 4200 (Agilent Technologies, Palo Alto, CA, USA). Gene profiles were established by Affymetrix Clariom™ S assay (Thermo Fisher Scientific). RNA labeling, processing, and hybridization were performed according to manufacturer’s standard protocols; microarrays were scanned with GeneChip Scanner 3000 7G Array (Thermo Fisher Scientific) and data were obtained using Affymetrix GeneChip Command Console Software (AGCC).

Raw Affymetrix CEL files were pre-processed using the frozen robust multi-array average (RMA) algorithm [48] from Bioconductor. Data pre-processing was performed using the RMA [49] function implemented in the oligo package. Platform annotation was obtained from Bioconductor annotation package clariomshumantranscriptcluster.db. Multiple probes mapping to the same gene symbol were collapsed using the collapseRows function of the WGCNA package with the “MaxMean” method.

Differential expression analysis was carried out using the standard linear modeling approach implemented in the limma package [50] comparing treated cell lines (Vemurafenib 20 µM or Dabrafenib 2 µM) versus DMSO. Nominal *p*-values were corrected for multiple testing using the Benjamini–Hochberg false discovery rate (FDR) [51]. Differentially expressed genes were selected according to absolute fold-change |(FC)| ≥ 2 and false discovery rate (FDR) < 0.05.

### 4.7. Functional Enrichment Analysis

Gene set enrichment analysis (GSEA) was performed in pre-ranked mode using the fgsea Bioconductor package [52] comparing treated cell lines versus DMSO control samples. Genes were ranked using the t-statistic derived from the differential expression analysis with limma. The number of permutations was set to 10,000 and gene sets with fewer than 15 or more than 500 genes were filtered out. Significant gene sets were selected according to FDR < 0.05 and graphed using ggplot2 R package. Functional category was then manually assigned to biological super category according to the Hallmarks gene sets Process Categories [25] and reported as a pie chart.

### 4.8. Gene Expression Scores Related to TCGA Derived Gene Signatures

Three gene signatures derived from TCGA study on PTCs [3] were tested: a MAPK output gene set comprising 52 genes; a thyroid differentiation (TD) gene set comprising 16 genes; and a BRAF-RAS signaling gene set comprising 71 genes. The corresponding gene lists are available in Table S2.

TD scores were calculated as mean of log2-transformed and median-centered expression across samples as previously described [3]; the same approach was applied for MAPK output score as previously reported [12]. The BRAF-RAS score was calculated as reported by Rusinek et al. [10]. An additional score based on the 71-gene signature related to BRAF-RAS signaling was computed by singscore [53] which implements a simple single-sample gene-set scoring method that scores individual samples for quantifying concordance between sample transcriptomes and selected molecular signatures.

### 4.9. Unsupervised Analysis of BCPAP Cell Lines and TC Tumors

A gene profile series of 29 human thyroid cancer tissues previously established by our laboratory [8] and available on Gene Expression Omnibus repository with accession number GSE104005 was tested.

To compute similarity between BCPAP cell lines and TC tissues expression data, we first reduced batch effects by selecting common genes between the two datasets (*n* = 16,346), then these genes were standardized across the samples of each dataset by z-score calculation. We merged the two datasets and applied unsupervised analyses. Euclidean distance and Ward linkage metrics were used for the hierarchical clustering and then graphed with ComplexHeatmap R package. Principal component analysis was also computed with the prcomp R function to cluster the samples according to the BRAF/RAS-signaling signature.

### 4.10. Western Blot Analysis

Total protein extraction, SDS PAGE, and Western blot analysis were performed as previously described [54]. The following primary antibodies were used: pMEK1/2 (phosphor-MEK1/2 Ser217/221) #9121, MEK1/2 (L38C12) #4696, pERK1/2 (phospho-ERK1/2 Thr202/Tyr204) #9101, ERK1/2 (L34F12) #4696 from Cell Signaling Technology Inc (Danvers, MA, USA); Vinculin #V9131 from Sigma-Aldrich was included as protein loading control.

## Data Availability

Raw and pre-processed gene expression data presented in this study are available in the NCBI Gene Expression Omnibus (GEO) repository with the accession number GSE171483.

## Supplementary Materials

The following are available online at https://www.mdpi.com/1422-0067/22/11/5744/s1. Figure S1: BRAFV600E mutation testing in thyroid cancer cell lines; Figure S2: Gene set enrichment analysis with Hallmarks gene set collection in BCPAP cells treated with BRAF inhibitors; Figure S3: GSEA on Hallmark KRAS signaling gene sets in BCPAP cells treated with BRAF inhibitors; Figure S4: Unsupervised analysis using the BRAF-RAS signaling gene signature in BCPAP cells and in thyroid cancer tissues; Figure S5: Alternative MAPK pathway activation upon BRAF inhibitors treatment in 8505C and K1 cell lines; Table S1: Characterization of the thyroid cancer derived cell lines used in the present study; Table S2: Lists of genes included in the TCGA-derived gene signature related to thyroid cancer.
