# Supplementary material for: BRAF Inhibitors Induce Feedback Activation of RAS Pathway in Thyroid Cancer Cells
Source: Int J Mol Sci. 2021 May 27;22(11):5744. doi: 10.3390/ijms22115744 (PMC8198461; doi:10.3390/ijms22115744)
Supplement: Supplementary file 1 [file ijms-22-05744-s001.zip › ijms-1222679-supplementary.pdf]

SUPPLEMENTARY FIGURES

Supplementary Figure 1

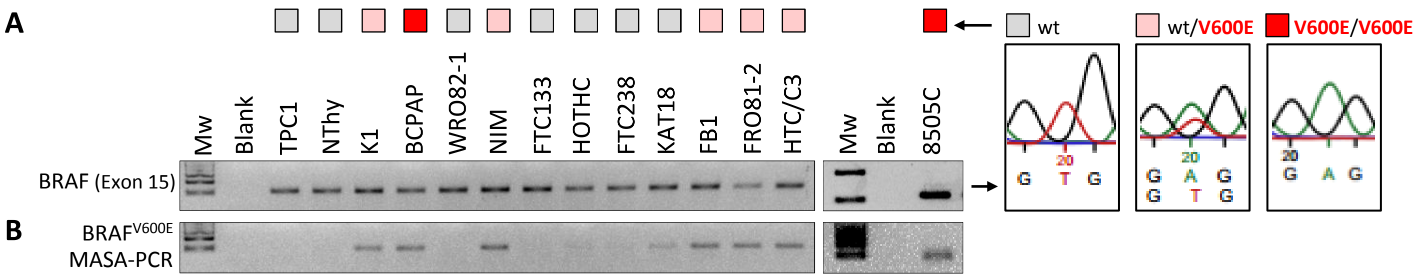

Figure S1. BRAF^{V600E} mutation testing in thyroid cancer cell lines.

A - B. BRAF^{V600E} mutation was assessed in 13 thyroid cancer (TC) cell lines and in the non-neoplastic thyroid (NT) control Nthy cells by BRAF exon 15 specific PCR followed by automated Sanger sequencing and by mutant-allele-specific-amplification(MASA)-PCR method. PCR products were resolved by ethidium bromide stained agarose gel electrophoresis. On the right three representative electropherograms obtained for BRAF wt or mutated (hetero- or homo-zygous) cell lines.

Abbreviations: Mw, molecular weight markers 100 base pairs; Blank, PCR mix reaction negative control; wt, wild-type;

Supplementary Figure 2

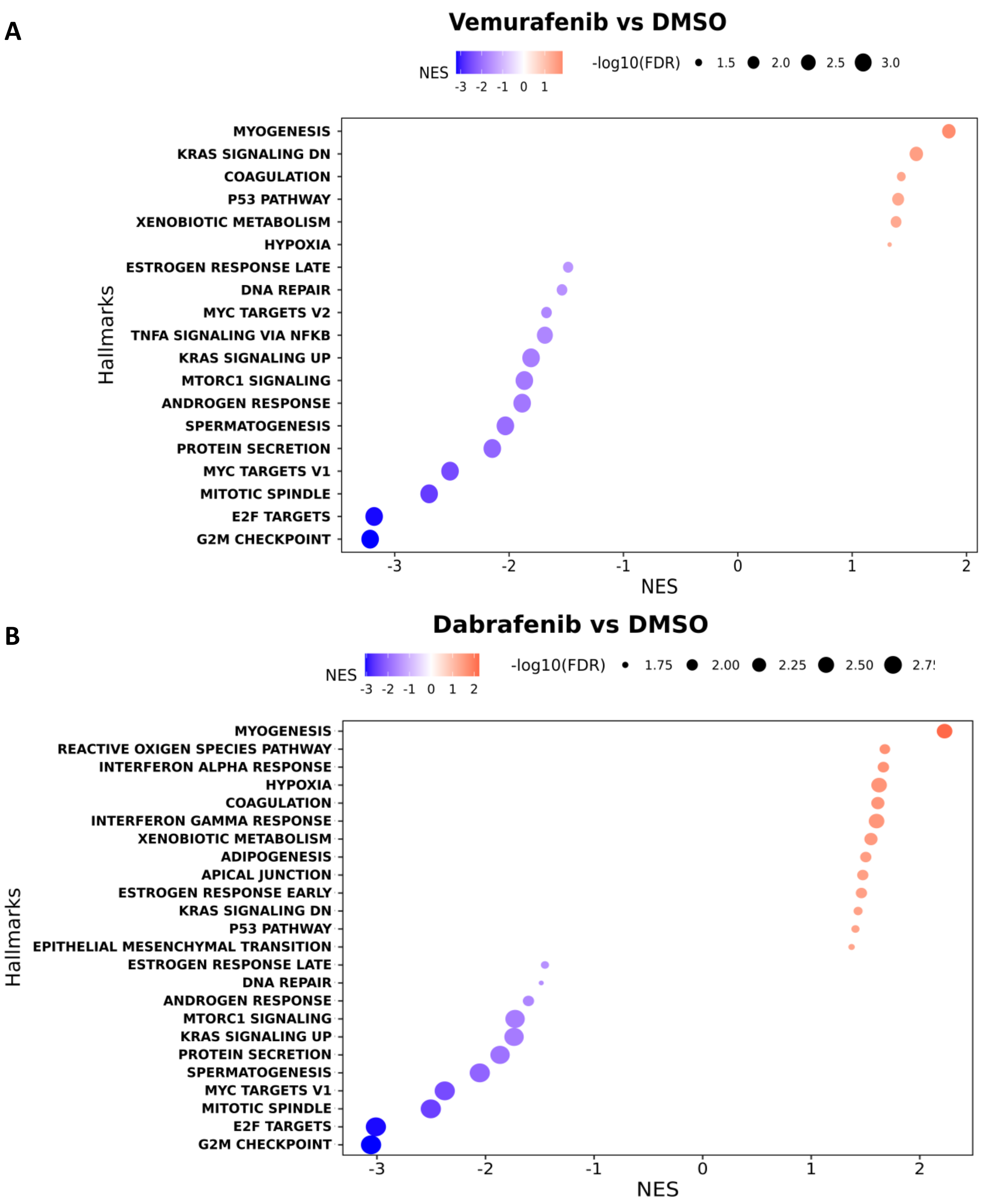

Figure S2. Gene set enrichment analysis with Hallmarks gene set collection in BCPAP cells treated with BRAF inhibitors. Dot plots showing all the significantly enriched gene sets (FDR < 0.05) from GSEA Hallmarks collection in BCPAP cells treated with Vemurafenib (**A**) or Dabrafenib (**B**) versus control cells treated with DMSO. The red-to-blue colored-scale bar represents the normalized enrichment score (NES). The point size indicates the magnitude of the statistical significance expressed as –log10 of the FDR.

Supplementary Figure3

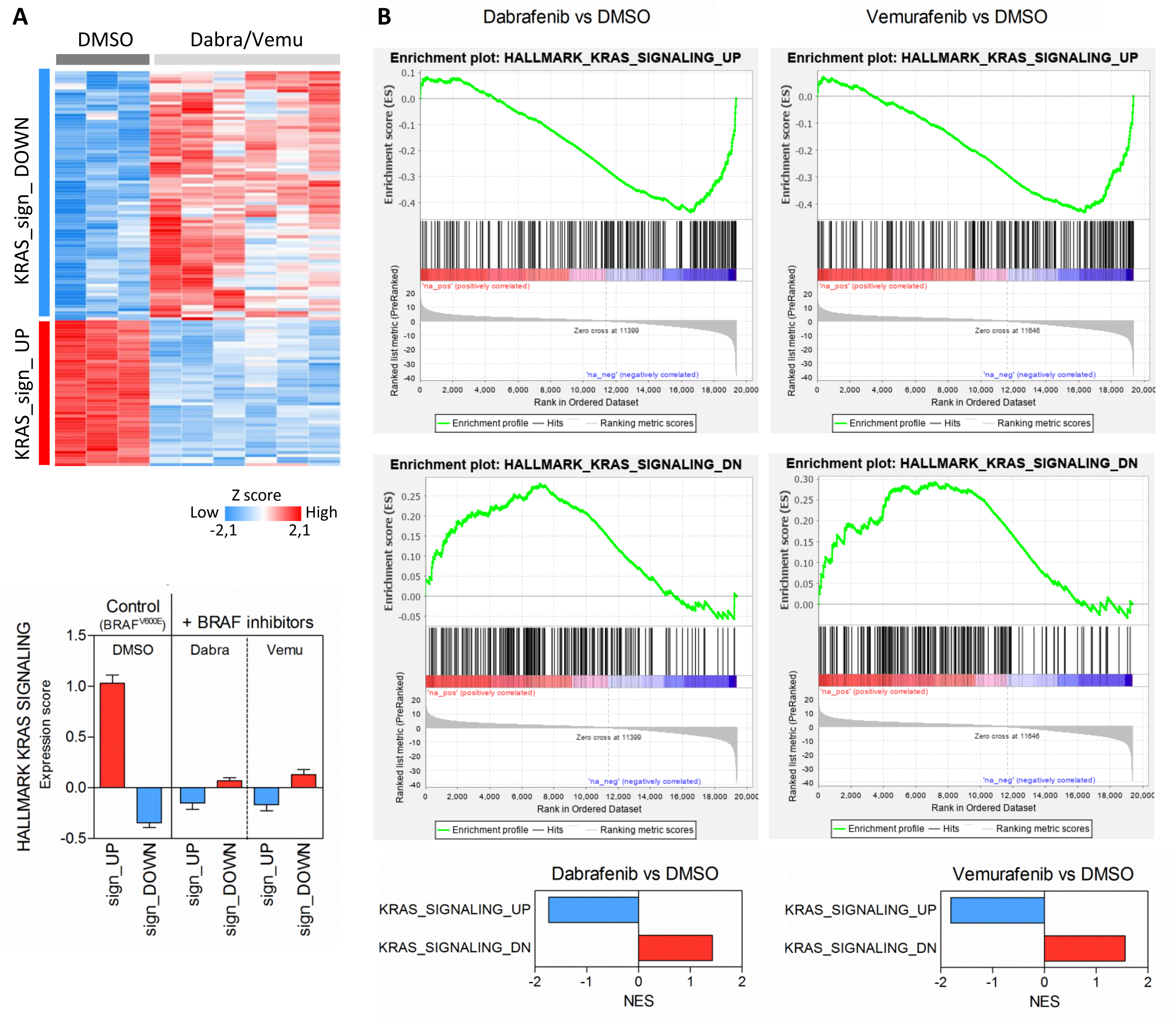

Figure S3. GSEA on Hallmark KRAS signaling gene sets in BCPAP cells treated with BRAF inhibitors.

A. Heatmap showing the expression of KRAS_SIGNALING_UP and KRAS_SIGNALING_DN gene in BCPAP cells; the leading-edge subset of genes common across the two treatment are specifically showed. Below the corresponding expression score calculated as mean of log2-transformed and median-centered gene levels; separate scores were calculated for the KRAS_SIGNALING_UP (sign_UP) and KRAS_SIGNALING_DN (sign_DOWN) gene sets. **B.** Enrichment plots from GSEA conducted in BCPAP cells treated with BRAF inhibitors (Dabrafenib or Vemurafenib) versus control cells (DMSO). The KRAS_SIGNALING_UP and the KRAS_SIGNALING_DN gene sets of Hallmarks collection are specifically showed. The red-to-blue color bar shows the ranking of the genes of each dataset from up- to down-regulated in Dabrafenib or Vemurafenib, lower and upper panel respectively. The vertical black bars indicate the position of BCPAP cell genes along the ranked gene list. The green line shows the running enrichment score (ES) along the ranked gene list. The corresponding normalized enrichment scores (NES) are reported below the enrichment plots.

Supplementary Figure 4

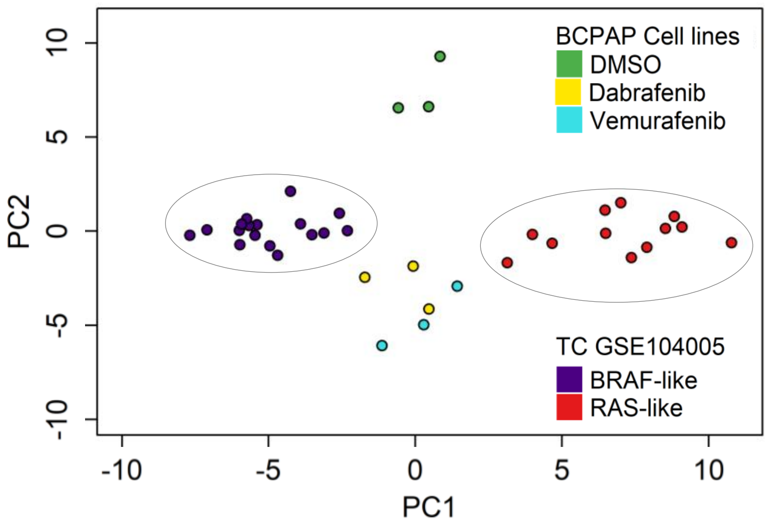

Figure S4. Unsupervised analysis using the BRAF-RAS signaling gene signature in BCPAP cells and in thyroid cancer tissues. Principal component analysis (PCA) using the BRAF/RAS-like gene signature calculated in the merged dataset with BCPAP cells and in human thyroid cancer (TC) tissues derived from the dataset GSE104005. For TC tissues the feature relative to the BRAF- or RAS-like subtype has been previously established and here showed; TC tissues are highlighted by circles

Supplementary Figure 5

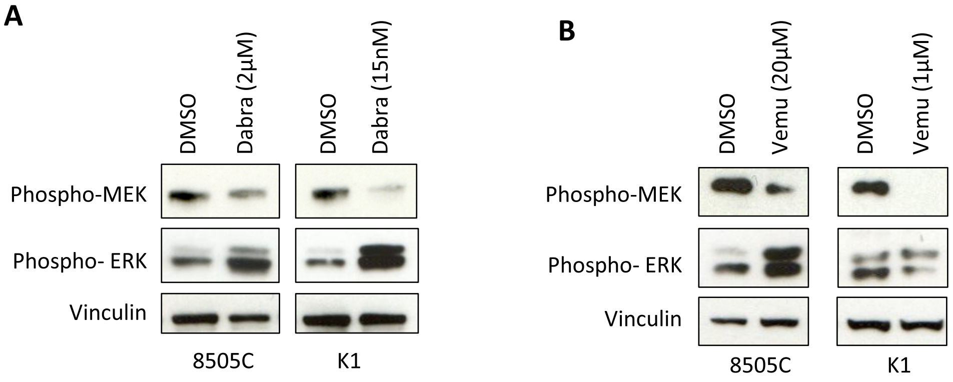

Figure S5. Alternative MAPK pathway activation upon BRAF inhibitors treatment in 8505C and K1 cell lines. Western blot analysis in the thyroid cancer derived cell line 8505C and K1 treated with Dabrafenib (**A**) or Vemurafenib (**B**) for 48h compared to control cells treated with vehicle DMSO. Vinculin included as protein loading control.

Supplementary Table S1.

Characterization of the thyroid cancer derived cell lines used in the present study

Tissue type	Cell line	STR profile (reference)	BRAF mutation (this study)	BRAF mutation (reference)
NT	Nthy-ori3-1	CVCL_2659	wt	[3]
PTC	K1	CVCL_2537	**V600E**/wt	[3]
PTC	NIM1	CVCL_5344 [a]	**V600E**/wt	[4]
PTC	BCPAP	CVCL_0153	**V600E/V600E**	[3]
PTC	TPC1	CVCL_6298	wt	[3]
FTC	WRO82-1	[1] [b]	wt	[5]
FTC	FTC133	CVCL_1219	wt	[3]
FTC	FTC238	CVCL_2447	wt	[3]
ATC	8505C	CVCL_1054	**V600E/V600E**	[3]
ATC	FB1	CVCL_A603 [a]	**V600E**/wt	This study [c]
ATC	FRO81-2	[2] [b]	**V600E**/wt	[5]
ATC	HTC/C3	CVCL_1295	**V600E**/wt	[3]
ATC	HOTHC	CVCL_8708	wt	This study [c]
ATC	KAT18	CVCL_6303	wt	[3]

Abbreviation: NT, non-neoplastic thyroid; PTC, papillary thyroid carcinoma; FTC, follicular thyroid carcinoma; ATC, anaplastic thyroid carcinoma; wt, wild type; CVCL, Cellosaurus ID (reference cell line ID)

[1] Xu, X. *et al.* Endocr Relat Cancer 2012, 19, 167-179;

[2] Shimamura, M. *et al.* Endocr J 2014, 61, 481-490;

[3] Landa, I. *et al.* Clin Cancer Res 2019, 25, 3141-3151;

[4] Degl'Innocenti, D. *et al.* PLoS One 2010, 5(9), e12701;

[5] Namba, H. *et al*. J Clin Endocrinol Metab 2003, 88, 4393-4397;

[a] No STR profiles are reported for these cell lines. The STR profiles identified in the present study are unique and different from those of other cell lines. NIM1: Amelogenin, X; CSF1PO, 9 and 10; D13S317, 10 and 14; D16S539, 10 and 12; D21S11, 30; D5S818, 9 and 10; D7S820, 11; TH01, 9; TPOX, 8; vWA, 16 and 20. FB1: Amelogenin, X; CSF1PO, 11 and 12; D13S317, 14; D16S539, 10 and 11; D21S11, 29 and 30; D5S818, 11and 12; D7S820,10; TH01, 7and 9; TPOX, 8 and 9; vWA, 16 and 17.

[b] Conflicting data on STR profiles for these cell lines; the STR profiles identified in the present study matched with those reported in the indicated reference.

[c] No previous reports on BRAF mutation assessment in these cell lines.

Supplementary Table S2.

Lists of genes included in the TCGA-derived gene signature related to thyroid cancer

MAPK output	Thyroid differentiation	BRAF-RAS signaling	
ARID5A	TSHR	ANKRD46	RUNX2
B4GALT6	DUOX1	CYB561	SDC4
BRIX1	TG	GNA14	SEL1L3
BYSL	THRB	HGD	SFTPB
CCND1	DIO1	KATNAL2	SLC35F2
CD3EAP	DIO2	KCNAB1	SOX4
CHSY1	DUOX2	KCNIP3	SPOCK2
DDX21	FOXE1	LGI3	STK17B
DUSP4	GLIS3	MLEC	SYT12
DUSP6	THRA	NQO1	TACSTD2
EGR1	SLC5A5 (NIS)	SFTPC	TBC1D2
ELOVL6	SLC26A4	SLC4A4	TGFBR1
ETV4	SLC5A8	SORBS2	TMEM43
ETV1	NKX2-1	ABTB2	FAM176A (EVA1A)
ETV5		AHR	TM7SF4 (DCSTAMP)
FOS		ANKLE2	PVRL4 (NECTIN4)
FOSL1		ANXA1	DTX4
GEMIN4		ARNTL	ANXA2P2*
GNL3		ASAP2	FLJ23867*
GPR3		BID	
GTF2A1L		CDC42EP1	
GTPBP4		COL8A2	
HMGA2		CREB5	
HYDIN		CTSC	
IER3		CYP1B1	
CXCL8 (IL8)		DUSP5	
LIF		ETHE1	
MAFF		FAM20C	
MAP2K3		FCHO1	
MYC		FN1	
NOP16		FSTL3	
PHLDA2		GABRB2	
PLK3		GBP2	
POLR1C		ITGA3	
POLR3G		ITGB8	
PPAT		KCNN4	
RRS1		LAMB3	
SEMA6A		LLGL1	
SH2B3		LY6E	
SLC1A5		MDFIC	
SLC4A7		MET	
SPRED2		PDE5A	
SPRY2		PDLIM4	
SPRY4		PLCD3	
TNC		PLEKHA6	
TNFRSF12A		PNPLA5	
TSR1		PPL	
WDR3		PRICKLE1	
YRDC		PROS1	
KIR3DL2 *		PTPRE	
PPAN *		RASGEF1B	
PYCRL*		RUNX1	

* These genes were not available for BCPAP cells gene profiles and were excluded from score computation.
Alternative gene alias are showed in brackets.
